# Diagnostic Accuracy of Liver and Spleen Stiffness in Magnetic Resonance Elastography for the Detection of Gastroesophageal Varices: A Systematic Review and Meta-Analysis

**DOI:** 10.3390/diagnostics13233527

**Published:** 2023-11-24

**Authors:** You Zheng, Kaifeng Huang, Xiaojing He, Tianwu Chen, Wei Jiang, Jun Zhou, Yangyang Liu, Dajing Guo

**Affiliations:** 1 Department of Radiology, The Second Affiliated Hospital of Chongqing Medical University, No. 74 Linjiang Rd., Yuzhong District, Chongqing 400010, China; zhengyou@hospital.cqmu.edu.cn (Y.Z.); he_xiaojing@hospital.cqmu.edu.cn (X.H.); tianwuchen_nsmc@163.com (T.C.); 302947@hospital.cqmu.edu.cn (W.J.); zhoujun@hospital.cqmu.edu.cn (J.Z.); liuyy@cqmu.edu.cn (Y.L.); 2 Department of Ultrasound, The Cancer Hospital of Chongqing University, Chongqing 400044, China; 18582387828@163.com

**Keywords:** magnetic resonance elastography, liver stiffness, spleen stiffness, gastroesophageal varices, meta-analysis

## Abstract

Background: The aim of this meta-analysis was to assess the performance of magnetic resonance elastography (MRE) in detecting gastroesophageal varices (GEV) in patients with chronic liver disease (CLD). Methods: A literature search in English and Chinese databases such as PubMed, EMBASE, Cochrane Library, Web of Science, and China National Knowledge Infrastructure was conducted. The pooled sensitivity, specificity, positive likelihood ratio (PLR), negative likelihood ratio (NLR), diagnostic odds ratio (DOR), and area under the curve (AUC) of the summary receiver-operating characteristic (SROC) curve with a 95% CI were calculated. A quality analysis of the included study was conducted using the QUADAS-2 tool, and a meta-analysis was performed using Stata16. The clinical practical value of MRE in detecting GEV was evaluated using the Fagan plot. Heterogeneity across studies was explored through meta-regression and subgroup analyses. Results: A total of nine relevant articles that compared liver stiffness (LS) or spleen stiffness (SS) using MRE with esophagogastroduodenoscopy (EGD) to detect the existence of GEV were identified. The pooled summary sensitivity, specificity, PLR, NLR, and DOR of LS or SS for the detection of GEV were 81% (95% CI: 74%, 87%), 72% (95% CI: 62%, 80%), 2.89 (95% CI: 2.12, 3.94), 0.26 (95% CI: 0.19, 0.36), and 10.91 (95% CI: 6.53, 18.24), respectively. The year of publication, study design, and MR equipment are the sources of heterogeneity. There was no significant difference in the publication bias (*p* > 0.05). Conclusions: Based on these findings, MRE demonstrates good diagnostic accuracy for detecting GEV in patients with CLD.

## 1. Introduction

Chronic liver disease (CLD) is a public health concern across the world. CLD and cirrhosis are the 11th major cause of death around the world, resulting in an estimated 2 million deaths annually, with half of them owing to complications of cirrhosis [1,2]. The global prevalence of liver cirrhosis has risen from 71 million in 1990 to more than 122 million in 2017 [1]. The incidence of gastroesophageal varices (GEV) has been reported in half of the number of patients with cirrhosis, and variceal hemorrhage accounts for around 5–15% of varices episodes [3]. Gastroscopy is the current “gold standard” for the diagnosis and grading of GEV, although it is invasive, uncomfortable, expensive, and operator-dependent, which may bring about bleeding with lots of restrictive factors. Furthermore, repeated endoscopy may be hard to accept by those who are in ongoing longer-term follow-up. As such, we are in urgent need of alternative non-invasive reliable and accurate methods to evaluate GEV. Imaging techniques, including transient elastography (TE), shear wave elastography (SWE), FibroScan (FS), multi-slice spiral CT, and magnetic resonance imaging (MRI), have been used to evaluate GEV so far. TE and magnetic resonance elastography (MRE) are the available imaging tools for predicting diffuse liver disease. Because of the limitations of ultrasonic imaging mechanisms, TE has inherent disadvantages. It is highly operator-dependent, less reliable for deep organs, and has increased variability in obese patients [4]. However, MRE has been implemented successfully in a variety of patient populations, including those with ascites, obesity, and unconventional hepatic anatomy [5]. MRE has been shown to offer a greater degree of diagnostic accuracy compared to TE [6]. MRE is a new phase contrast-based diagnostic imaging modality. It differs from traditional MRI, as it generates shear waves within the organs by utilizing mechanical or acoustic modalities, and then quantitatively measures the internal stiffness distribution of tissues [7]. MRE can be regarded as a quantitative and non-invasive technique. It is convenient, safe, and comfortable for the patients. The clinical importance of it has been increasing day by day. Hepatic MRE is easy to carry out and can be easily repeated in a short period. Studies have shown that liver stiffness (LS) or spleen stiffness (SS) may be interrelated with GEV [8,9]. MRE has been introduced into clinical practice, and there have been several studies that have investigated the specific relationships between LS or SS and the presence of GEV [10,11]. Considering that the results and quality have considerable variability across different published studies, the diagnostic value and feasibility of MRE in detecting GEV remain elusive. 

Hence, the primary purpose of the present review is to perform a systematic review and structured meta-analysis of previous eligible studies to assess the efficacy of MRE for screening and diagnosing GEV and to attempt to provide more comprehensive theoretical support for quantitative monitoring in real time and a clinical therapeutic strategy for cirrhotic patients with GEV.

## 2. Materials and Methods

### 2.1. Search Strategy

Each part of this article was written while taking into account the PRISMA guidelines [12]. To identify relevant clinical studies evaluating MRE for the diagnosis of GEV in patients with CLD, a systematic literature search in English and Chinese electronic databases including PubMed, EMBASE, Cochrane Library, Web of Science, and China National Knowledge Infrastructure was conducted up until 9 December 2022. The key terms used for the search included “Esophageal and Gastric Varices” and “Magnetic Resonance Elastography”. We searched for related articles as much as possible to evaluate the text. All references that were retrieved by applying the literature retrieval strategy were initially screened by title, and then by abstract, and finally, by full text.

### 2.2. Eligibility Criteria

The major inclusion criteria of our meta-analysis are described below: (1) Endoscope was used as a diagnostic “gold-standard” for the identification of GEV in CLD patients. (2) LS or SS was measured via MRE. (3) Studies had to contain enough necessary data to allow for the test performance be calculated, including true positive (TP), false positive (FP), true negative (TN), and false negative (FN) values based on the best statistical cutoff values of MRE for the detection of GEV. (4) The sample size should be no less than 20 patients to ensure trustworthiness. Animal studies, ex vivo studies, case reports, meetings, review articles, and duplicated studies were excluded.

Two investigators (ZY and HKF) independently extracted data from each study. If there were any different opinions between 2 researchers, the differences were resolved through discussion.

### 2.3. Data Extraction and Quality Assessment

Two independent reviewers (ZY and HXJ) screened the literature, extracted information, and appraised the methodological quality. Any discrepancy between 2 researchers was resolved via consensus, with the final judgment made by a 3rd experienced radiologist (GDJ). For the studies that were eligible for this systematic review, the primary data were abstracted below: first author’s name, location, year of publication, study period, study design, etiology, sample size, proportion of males, age, body mass index (BMI), Child–Pugh score, the time intervals of EGD and MRE, cut-off point, sensitivity, specificity, TP, FP, TN, and FN. When both the validation cohort and training cohort are provided in one study, we extracted data from the literature solely from the validation cohort. A methodological quality assessment was performed, adapting to the modified Quality Assessment of Diagnostic Accuracy Studies (QUADAS-2) [13]. 

### 2.4. Statistical Analysis

The pooled sensitivity (the proportion of those with the disease who have true positive results), specificity (the proportion of those without the disease who have true negative results), positive likelihood ratio (PLR; the sensitivity divided by the false negative rate), negative likelihood ratio (NLR; the FN rate divided by the specificity), positive predictive value (PPV), negative predictive value (NPV), diagnostic odds ratio (DOR), and area under the curve (AUC) of the summary receiver-operating characteristic (SROC) curve with a 95% CI were calculated using a bivariate random-effects model. The interstudy statistical heterogeneity of all diagnostic parameters was assessed with a visual inspection of forest plots, and then statistic calculations were performed with the Cochrane’s Q-test and inconsistency index (I^2^). *p* < 0.05 or I^2^ > 50% was generally considered to indicate the existence of heterogeneity across the included studies, and a larger I^2^ value signified a higher degree of heterogeneity. Univariate meta-regression analysis and subgroup analysis will be performed to explore potential sources of heterogeneity. Fagan diagram was used to evaluate the clinical utility of MRE for diagnosing GEV. Publication bias was further tested using Deeks’ funnel plot to guarantee the validity of results. The quality analysis of the included study was evaluated using the QUADAS-2 tool, and the meta-analysis was performed with Stata16.

## 3. Results

### 3.1. Literature Selection

**Figure 1:**
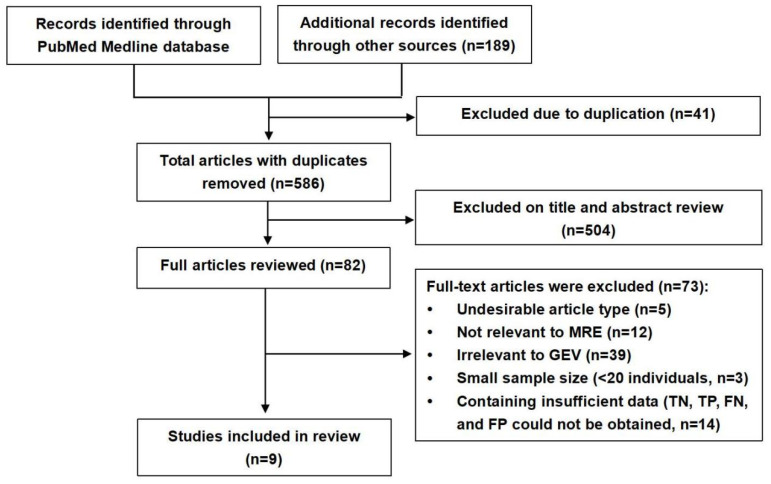
Flowchart for literature retrieval and research selection.

**Figure 2:**
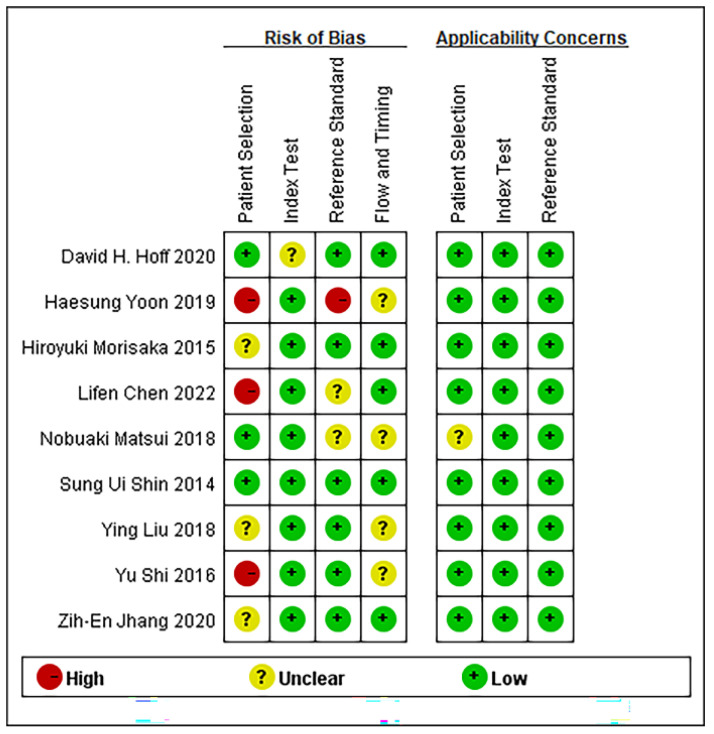
Quality of methodology of included studies based on QUADAS-2 tool criteria [14,15,16,17,18,19,20,21,22].

**Figure 3:**
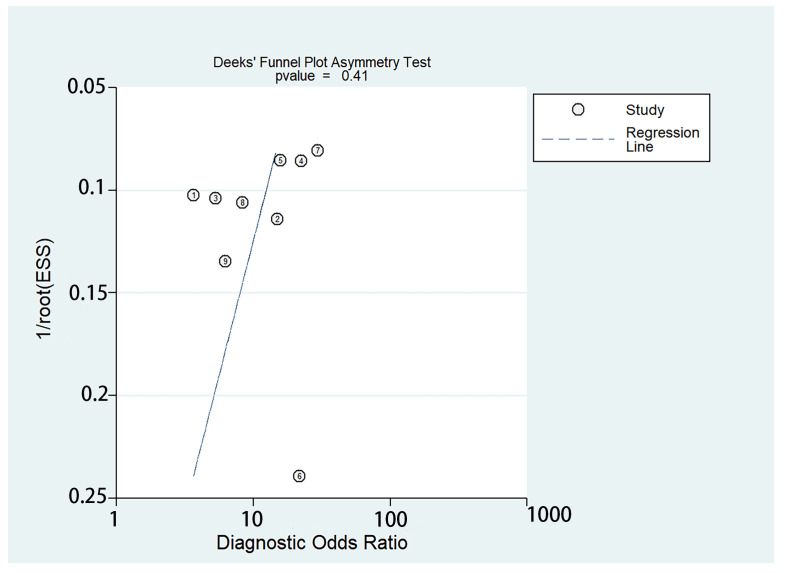
Pooled results of the included studies to investigate the potential publication bias, presented in a Deeks’ funnel plot [14,15,16,17,18,19,20,21,22].

Based on the established search strategy, 627 studies that potentially met the inclusion criteria were retrieved, in which 41 pieces of duplicate reports were excluded. We preliminarily excluded 328 studies by reading the titles. Through reading the abstracts, 176 studies that were irrelevant to our study were excluded. Next, we examined the full texts of the remaining 82 studies cautiously. Of these, 73 articles were excluded due to an undesirable article type (*n* = 5), because they were not relevant to MRE (*n* = 12), irrelevant to GEV (*n* = 39), had a small sample size (fewer than 20 individuals, *n* = 3), and contained insufficient data (TN, TP, FN, and FP could not be obtained, *n* = 14). Ultimately, nine articles that fulfilled the inclusion criteria were included. A flow diagram of the literature search and study selection is presented in Figure 1. The detailed relevant characteristics of the nine included studies are summarized in Table 1, and Figure 2 lists the quality evaluation of the included studies. The four domains were identified, including patient selection, reference standard, index test, and flow and timing. Furthermore, we also assessed the first three domains for concerns regarding applicability. A summary of the proportion of all trials that were at high, low, or unclear risks of bias is shown. Meanwhile, Deek’s funnel plot asymmetry test confirmed that there was no publication bias (*p* =  0.41) among the included studies (Figure 3). A total of 979 patients were included. Seven original studies were retrospective, and two were prospective in design.

**Table 1:** Study characteristics.

Study	Location	Duration	Study Design	MR	Etiology	Number of Patients	Gender (Male)	TP	FP	FN	TN	Number of GEV	BMI	Mean Age	Child–Pugh Score, A/B/C
Jhang et al. (2020) [14]	Taiwan, China	2016.1–2018.9	retrospective	1.5 T	HBV, HCV, alcohol, etc.	102	68	42	13	22	25	64	NR	61.8 ± 12.0	NR
Hoff et al. (2020) [15]	USA	2018.6–2018.12	prospective	3.0 T, 1.5 T	HBV, HCV, NAFLD, etc.	78	NR	27	9	7	35	34	NR	NR	NR
Morisaka et al. (2015) [16]	Japan	2011.9–2012.3	retrospective	1.5 T	HBV, HCV, alcohol autoimmune hepatitis,	93	59	40	32	4	17	44	20.8 ± 7	69 ± 8	74/17/2
Matsui et al. (2018) [17]	Japan	2013.4–2016.12	retrospective	3.0 T	NAFLD, HBV, HCV, PBC, alcohol	243	130	37	59	4	143	41	23.4 ± 1.32	66.8 ± 12.1	NR
Shin et al. (2014) [18]	Korea	2010.11–2012.3	prospective	1.5 T	HBV, HCV, PBC, alcohol, autoimmune hepatitis, etc.	139	102	67	17	11	44	78	NR	57.3 ± 10.5	NR
Yoon et al. (2019) [19]	Korea	2015.1–2016.12	retrospective	3.0 T	Patients with biliary atresia that underwent the Kasai operation	22	10	5	3	1	13	6	17.1	10	NR
Chen et al. (2022) [20]	China	2019.2–2020.2	retrospective	3.0 T	NR	154	NR	67	11	13	63	80	17.1	NR	NR
Yu Shi et al. (2016) [21]	China	2013–2015	retrospective	3.0 T	HBV, HCV, alcohol, NASH, autoimmune hepatitis	89	62	34	13	10	32	44	NR	NR	68/17/4
Ying Liu et al. (2018) [22]	China	2015.12–2016.12	retrospective	3.0 T	HBV, HCV, etc.	59	36	24	5	13	17	37	NR	43.2 ± 11.2	NR

MR, magnetic resonance; TP, true positive; FP, false positive; FN, false negative; TN, true negative; GEV, gastroesophageal varices; BMI, body mass index; HBV, hepatitis B virus; HCV, hepatitis C virus; NAFLD, non-alcoholic fatty liver disease; NASH, non-alcoholic steatohepatitis; PBC, primary biliary cirrhosis; NR, not reported.

### 3.2. Accuracy of MRE for the Detection of GEV

**Figure 4:**
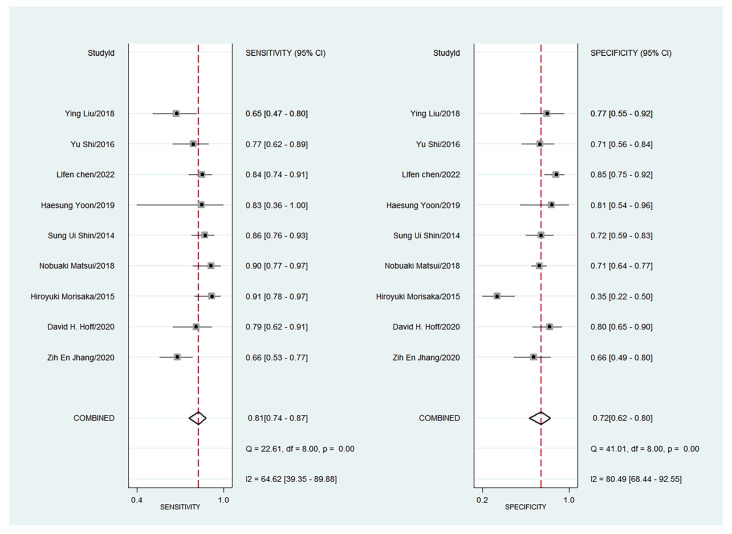
Forest plot of sensitivity and specificity for the liver or spleen stiffness for diagnosis of GEV (95% CI) [14,15,16,17,18,19,20,21,22].

**Figure 5:**
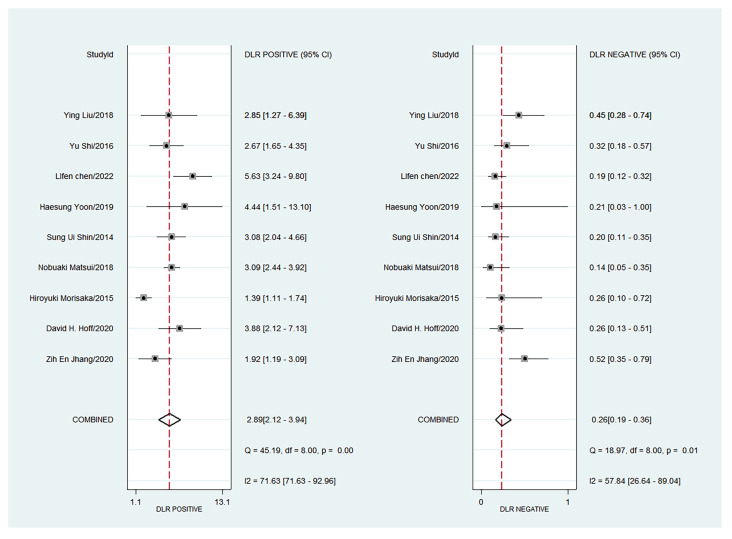
Forest plot of PLR and NLR for the liver or spleen stiffness for diagnosis of GEV (95% CI) [14,15,16,17,18,19,20,21,22].

**Figure 6:**
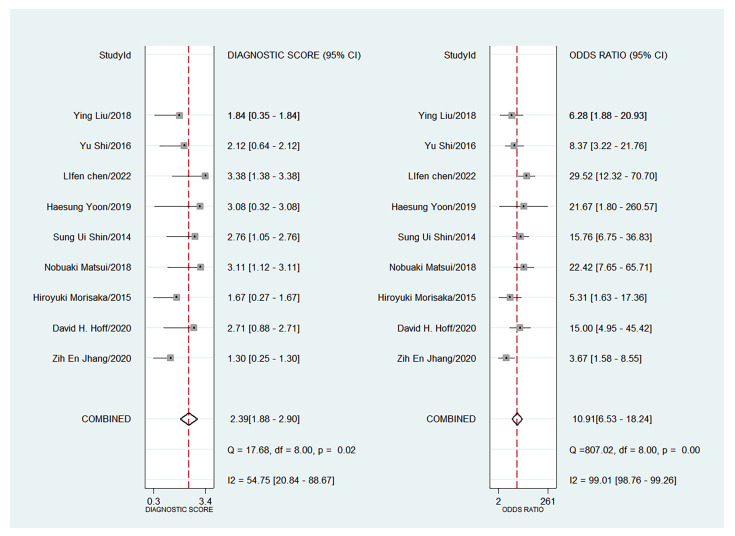
Forest plot of DOR for the liver or spleen stiffness for diagnosis of GEV (95% CI) [14,15,16,17,18,19,20,21,22].

**Figure 7:**
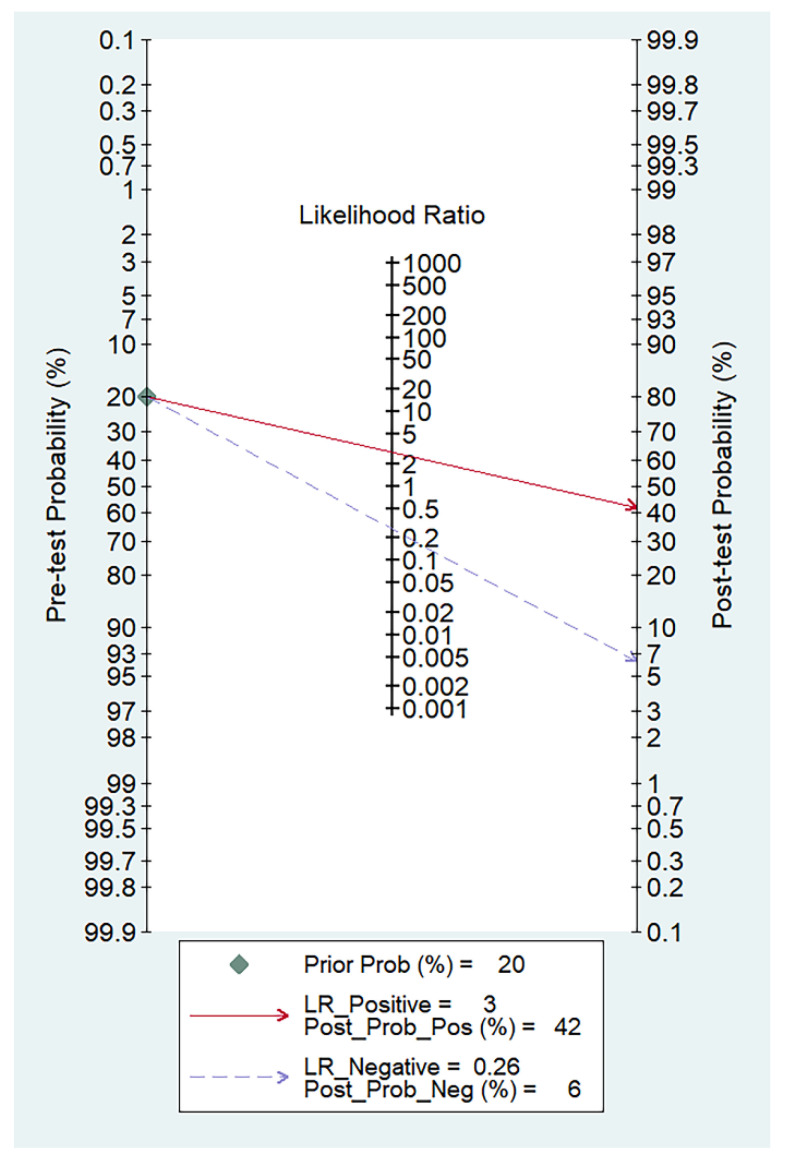
Fagan nomogram of liver or spleen stiffness for diagnosis of GEV.

**Figure 8:**
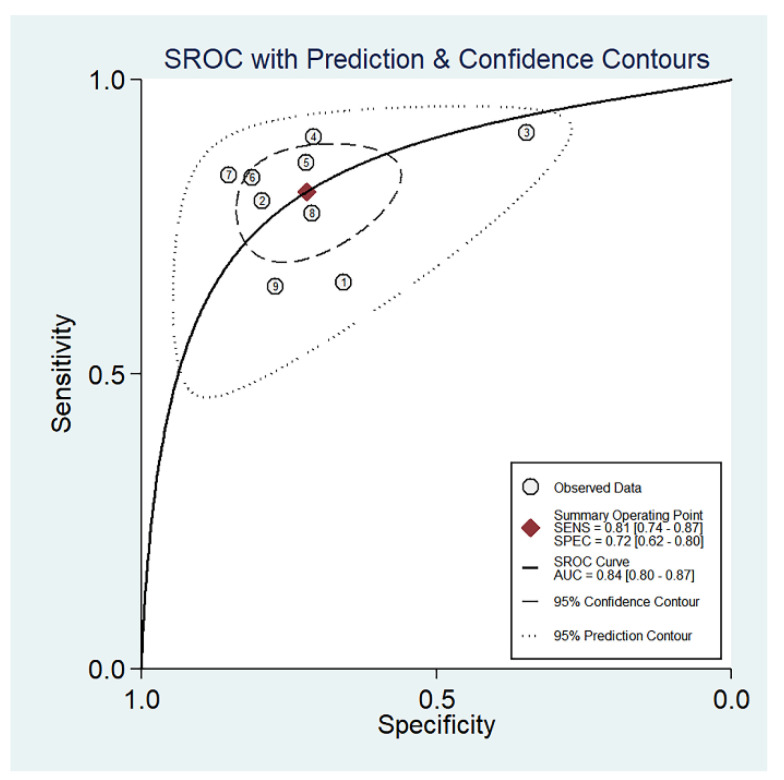
Summary receiver operating curve (SROC) for GEV.

The forest plots of sensitivity, specificity, PLR, NLR, and DOR of the nine studies are shown in Figure 4, Figure 5 and Figure 6. Among the nine included studies, GEV were present in 428 patients via endoscopic examination. The comparison revealed that the mean LS or SS values measured with MRE were significantly higher in the GEV group compared to the non-GEV group. The pooled summary sensitivity, specificity, PLR, NLR, and DOR of LS or SS for the detection of GEV were 81% (95% CI: 74%, 87%), 72% (95% CI: 62%, 80%), 2.89 (95% CI: 2.12, 3.94), 0.26 (95% CI: 0.19, 0.36), and 10.91 (95% CI: 6.53, 18.24), respectively. Figure 7 represents the Fagan nomogram for the likelihood ratios; given that the pre-test probability of correctly diagnosing GEV was 20%, the post-test likelihood was increased to 42%. Figure 8 illustrates the corresponding SROC curve with an AUC of 0.84 (95% CI, 0.80–0.87), which suggests high diagnostic efficiency. 

### 3.3. Meta-Regression Analysis

**Figure 9:**
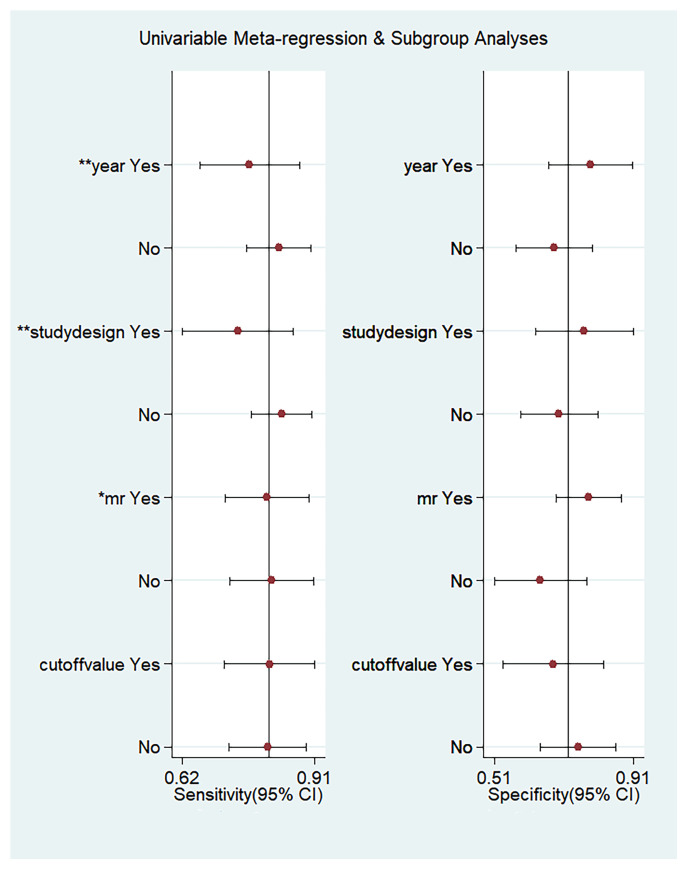
Univariable meta-regression and subgroup analyses. * *p* < 0.05, ** *p* < 0.01.

Due to significant heterogeneity, subgroup and meta-regression analyses were performed for the exploration of potential sources of heterogeneity factors. The meta-regression shows that the year of publication, study design, type of MR machine, or the cut-off value cannot explain the heterogeneity observed for the specificity. However, the year of publication, study design, and MR equipment can explain the heterogeneity observed for the sensitivity (Figure 9). All DOR (I^2^ = 99.0% and *p* < 0.01), PLR (I^2^ = 71.6% and *p* < 0.01), NLR (I^2^ = 57.8% and *p* < 0.01), sensitivity (I^2^ = 64.6% and *p* < 0.01), and specificity (I^2^ = 80.5% and *p* < 0.01) I^2^ values were above 50%.

## 4. Discussion

Acute bleeding from ruptured GEV is a serious clinical consequence of portal hypertension, which is the leading cause of the mortality of a patient. In the past, non-invasive evaluations of PV and GEV have always been one of the main interests for scholars in the world. During the development of liver cirrhosis and portal hypertension, the spleen and liver will enlarge and remodel progressively, including passive congestion, fibrogenesis, and vascular remodeling [23,24]. All of these changes caused an increase in LS and SS, and LS is strongly associated with PV and reflects the extra-hepatic hemodynamic changes. In terms of LS, it is clear that LS reflects the increased intrahepatic resistance. SS predicts the variceal formation resulting from splanchnic hemo-dynamics changes better than LS [8]. These lend reliable support to the physiological feasibility of LS and SS to detect GEV.

Numerous recent meta-analyses about the association between GEV and stiffness using ultrasonic elastography have emerged. However, there are no meta-analysis reports on the association between GEV and stiffness in MR. In terms of originality, this meta-analysis is the first paper to study the association between MRE and GEV. In this meta-analysis, a total of nine relevant articles that compared LS or SS (using MRE) with EGD to detect the existence of GEV were identified. The pooled sensitivity and specificity of LS/SS for detecting GEV were fairly good, and they were 81% (95% CI: 0.74–0.87) and 72% (95% CI: 0.62–0.80), respectively. Furthermore, the overall diagnostic performance, as evaluated by SROC (0.84), was also good, which suggested that MRE should be viewed as a valuable tool for prediction with a relatively high level of diagnostic efficiency. Tseng et al. assessed the diagnostic accuracy of CT for detecting GEV in 10 included studies, and they reported that the pooled specificity, sensitivity, PLR, NLR, DOR, and AUROC were 0.723, 0.896, 3.241, 0.143, 22.599, and 0.86, respectively, indicating that all of the summary indicators were similar to ours except for DOR [25]. Pu et al., Zhang et al., and Manatsathit et al. analyzed a total of 15, 24, and 45 studies, respectively, regarding the predictive accuracy of elastosonography for detecting the presence of EV, and the reported results were concordant with those of our study [26,27,28]. Cheng et al. assessed the diagnostic performance of TE in detecting the presence and size of esophageal varices in cirrhotic patients, and they found that an incorrect diagnosis would be possible in 24–44% of patients with a negative test result [29]. This suggests that TE might not be very accurate. Due to broader indications and a higher precision of MRE, it might be the preferred approach over TE in the future. And this technique might replace TE in some patient categories, such as patients with ascites and obesity.

We observed distinct heterogeneity among the selected studies, so a meta-regression analysis was performed to evaluate the origins of heterogeneities. In our study, the meta-regression analysis and subgroup analysis were based on the number of samples, year of publication, study area, study design, etiology, MR equipment, and cut-off value. The results showed that the heterogeneity came from the year of publication, study design, and MR equipment. The diagnostic value of prospective studies is significantly higher than that of retrospective studies, suggesting that a standardized experimental design, strict patient screening mechanism, and unified image processing methods are crucial to improve the diagnostic accuracy of GEV by MRE. Furthermore, the time span of the studies included in this study is large (around 10 years). During this period, MR examination techniques, post-processing methods, and diagnostic criteria have been greatly improved, which may be an important factor leading to high heterogeneity. MR equipment is another source of heterogeneity. The subgroup analysis showed that the accuracy of diagnosing GEV with MRE in 3.0T MR was higher than that of 1.5T MR; this might be related to the ability of MR devices with more outstanding performances to obtain higher-quality images. In addition, due to the limited number of included studies, we were unable to perform subgroup analyses on the control method, grouping standards, severe GEV ratio, etc. These factors may also contribute to the heterogeneity.

Previous studies have shown that AFRI and TE achieved a high diagnostic yield to predict EV, and when compared with LS, SS has more advantages in detecting EV [28,30]. They hypothesized that changes in LS halt when the portal vein pressure increases to a certain extent; however, relevant spleen parameters secondary to PV changes such as the spleen size and SS continue to change. Additionally, they suggested that the diagnostic performance of SS was significantly better than LS in detecting GEV, SS was superior to LS for the detection of EV with a higher sensitivity (0.90 vs. 0.85), LDOR (3.24 vs. 2.26), and AUC (0.899 vs. 0.817). In the current study, there was only a limited number of studies that evaluated the diagnostic accuracy of LS and SS in MRE for the detection of GEV. As a result, we did not perform a comparison between them.

There are several inevitable limitations in our meta-analysis as well. Firstly, this study includes only a limited number of articles, and the enrolled cases are limited. In the future, additional research is needed in multiple centers, and large-sample studies are needed for a more comprehensive evaluation. Secondly, notably different cutoff values were used in these studies, making it difficult to accurately define the standard diagnostic threshold value to predict GEV. It also makes our study fail to make a comparison of LS and SS on account of the absence of sufficient data from studies that performed LS and SS simultaneously on the same patient population. Likewise, the issue about the correlation between MRE grading and the variceal sizes cannot be carried out, either. Thirdly, additional unexplained heterogeneity persisted despite the meta-regression and subgroup analyses. The lack of longitudinal evaluations correlating MRE findings with clinical outcomes over time also limits the assessment of prognostic utility. Finally, the potential for publication bias is a concern in diagnostic test systematic reviews. Further large-scale studies validating standardized diagnostic cutoffs, directly comparing liver versus spleen stiffness, evaluating prognostic potential, and prospectively registering protocols could help strengthen the evidence base and overcome these limitations. In spite of a few deficiencies and limitations that exist in this study, the meta-analyses reported non-invasive clinical practice of MRE for the diagnosis of GEV.

In sum, this meta-analysis suggests that MRE achieves good accuracy in identifying GEV in liver cirrhosis patients. LS and SS may be beneficial to reduce the frequency of using invasive endoscopies to screen for GEV. Higher-quality studies and advanced data analysis technologies are needed to prove the prediction performance of MRE.

## Data Availability

Not applicable.

## References

[1] GBD 2017 Cirrhosis Collaborators (2020). The global, regional, and national burden of cirrhosis by cause in 195 countries and territories, 1990–2017: A systematic analysis for the Global Burden of Disease Study 2017. Lancet Gastroenterol. Hepatol..

[2] Asrani S.K., Devarbhavi H., Eaton J., Kamath P.S. (2019). Burden of liver diseases in the world. J. Hepatol..

[3] Lee E.W., Shahrouki P., Alanis L., Ding P., Kee S.T. (2019). Management options for gastric variceal hemorrhage. JAMA. Surg..

[4] Bastard C., Bosisio M.R., Chabert M., Kalopissis A.D., Mahrouf-Yorgov M., Gilgenkrantz H., Mueller S., Sandrin L. (2011). Transient micro-elastography: A novel non-invasive approach to measure liver stiffness in mice. World J. Gastroenterol..

[5] Venkatesh S.K., Yin M., Ehman R.L. (2013). Magnetic resonance elastography of liver: Clinical applications. J. Comput. Assist Tomogr..

[6] Kaplan J.M., Alexis J., Grimaldi G., Islam M., Izard S.M., Lee T.P. (2023). A comparison of magnetic resonance elastography (MRE) to biomarker testing for staging fibrosis in non-alcoholic fatty liver disease (NAFLD). Transl. Gastroenterol. Hepatol..

[7] Muthupillai R., Lomas D.J., Rossman P.J., Greenleaf J.F., Manduca A., Ehman R.L. (1995). Magnetic resonance elastography by direct visualization of propagating acoustic strain waves. Science.

[8] Colecchia A., Montrone L., Scaioli E., Bacchi-Reggiani M.L., Colli A., Casazza G., Schiumerini R., Turco L., Di Biase A.R., Mazzella G. (2012). Measurement of spleen stiffness to evaluate portal hypertension and the presence of esophageal varices in patients with HCV-related cirrhosis. Gastroenterology.

[9] Abe H., Midorikawa Y., Matsumoto N., Moriyama M., Shibutani K., Okada M., Udagawa S., Tsuji S., Takayama T. (2019). Prediction of esophageal varices by liver and spleen MR elastography. Eur. Radiol..

[10] Fraquelli M., Rigamonti C., Colombo M. (2012). Spleen stiffness measured by transient elastography accurately predicts esophageal varices in liver cirrhosis. Gastroenterology.

[11] Zhou H.H., Zhang Z.L., Zhang J., Sang L., Liu L.N., Gong X., Sun Y.Y., Zheng Y., Yu M. (2023). Performance of spleen stiffness measurement by 2D-shear wave elastography in evaluating the presence of high-risk varices: Comparative analysis of idiopathic portal hypertension versus hepatitis B virus. BMC. Med. Imaging.

[12] Page M.J., McKenzie J.E., Bossuyt P.M., Boutron I., Hoffmann T.C., Mulrow C.D., Shamseer L., Tetzlaff J.M., Akl A.E., Brennan S.E. (2021). The PRISMA 2020 statement: An updated guideline for reporting systematic reviews. BMJ.

[13] Whiting P.F., Rutjes A.W., Westwood M.E., Mallett S., Deeks J.J., Reitsma J.B., Leeflang M.M., Sterne J.A., Bossuyt P.M. (2011). QUADAS-2: A revised tool for the quality assessment of diagnostic accuracy studies. Ann. Intern. Med..

[14] Jhang Z.E., Wu K.L., Chen C.B., Chen Y.L., Lin P.Y., Chou C.T. (2021). Diagnostic value of spleen stiffness by magnetic resonance elastography for prediction of esophageal varices in cirrhotic patients. Abdom. Radiol..

[15] Hoffman D.H., Ayoola A., Nickel D., Han F., Chandarana H., Babb J., Shanbhogue K.P. (2020). MR elastography, T1 and T2 relaxometry of liver: Role in noninvasive assessment of liver function and portal hypertension. Abdom. Radiol..

[16] Morisaka H., Motosugi U., Ichikawa S., Sano K., Ichikawa T., Enomoto N. (2015). Association of splenic MR elastographic findings with gastroesophageal varices in patients with chronic liver disease. J. Magn. Reson. Imaging.

[17] Matsui N., Imajo K., Yoneda M., Kessoku T., Honda Y., Ogawa Y., Tomeno W., Fujisawa N., Misumi T., Kazumi K. (2018). Magnetic resonance elastography increases usefulness and safety of non-invasive screening for esophageal varices. J. Gastroenterol. Hepatol..

[18] Shin S.U., Lee J.M., Yu M.H., Yoon J.H., Han J.K., Choi B.I., Glaser K.J., Ehman R.L. (2014). Prediction of esophageal varices in patients with cirrhosis: Usefulness of three-dimensional MR elastography with echo-planar imaging technique. Radiology.

[19] Yoon H., Shin H.J., Kim M.J., Han S.J., Koh H., Kim S., Lee M.J. (2019). Predicting gastroesophageal varices through spleen magnetic resonance elastography in pediatric liver fibrosis. World J. Gastroenterol..

[20] Chen L.F., Yao C.G., Lan J., Huang L., Qin D.L., Ou Q., Wu X.Y., Huang S.X. (2022). A comparison between MR elastography and endoscopic ultrasonography for the diagnosis of esophageal and gastric varices in liver cirrhotic patients. Chin. Hepatol..

[21] Shi Y., Liu Y., Li Q.J., Li J.H., An H., Yu B., Guo Q.Y. (2016). Spin-echo echo planar imaging MR elastography in evaluation of gastroesophageal varices in liver cirrhosis. Chin. J. Med. Imaging Technol..

[22] Liu Y., Shi Y., Yu B., Li Q.J., Liu Y.Q., Wang M., Guo Q.Y. (2018). Comparison of MR elastography and dynamic contrast-enhanced imaging in evaluation on gastroesophageal varices with liver cirrhosis. Chin. J. Med. Imaging Technol..

[23] Bolognesi M., Merkel C., Sacerdoti D., Nava V., Gatta A. (2002). Role of spleen enlargement in cirrhosis with portal hypertension. Dig. Liver. Dis..

[24] Frulio N., Laumonier H., Balabaud C., Trillaud H., Bioulac-Sage P. (2009). Hepatic congestion plays a role in liver stiffness. Hepatology.

[25] Tseng Y.J., Zeng X.Q., Chen J., Li N., Xu P.J., Chen S.Y. (2016). Computed tomography in evaluating gastroesophageal varices in patients with portal hypertension: A meta-analysis. Dig. Liver Dis..

[26] Pu K., Shi J.H., Wang X., Tang Q., Wang X.J., Tang K.L., Long Z.Q., Hu X.S. (2017). Diagnostic accuracy of transient elastography (FibroScan) in detection of esophageal varices in patients with cirrhosis: A meta-analysis. World J. Gastroenterol..

[27] Zhang X., Chen C., Yan C., Song T. (2022). Accuracy of 2D and point shear wave elastography-based measurements for diagnosis of esophageal varices: A systematic review and meta-analysis. Diagn. Interv. Radiol..

[28] Manatsathit W., Samant H., Kapur S., Ingviya T., Esmadi M., Wijarnpreecha K., McCashland T. (2018). Accuracy of liver stiffness, spleen stiffness, and LS-spleen diameter to platelet ratio score in detection of esophageal varices: Systemic review and meta-analysis. J. Gastroenterol. Hepatol..

[29] Cheng F., Cao H., Liu J., Jiang L., Han H., Zhang Y., Guo D. (2018). Meta-analysis of the accuracy of transient elastography in measuring liver stiffness to diagnose esophageal varices in cirrhosis. Medicine.

[30] Hu X., Huang X.J., Hou J.H., Ding L., Su C.L., Meng F.K. (2021). Diagnostic accuracy of spleen stiffness to evaluate portal hypertension and esophageal varices in chronic liver disease: A systematic review and meta-analysis. Eur. Radiol..

